# PET Tracing of Biodistribution for Orally Administered ^64^Cu-Labeled Polystyrene in Mice

**DOI:** 10.2967/jnumed.120.256982

**Published:** 2022-03

**Authors:** Changkeun Im, Hyeongi Kim, Javeria Zaheer, Jung Young Kim, Yong-Jin Lee, Choong Mo Kang, Jin Su Kim

**Affiliations:** 1 Division of Applied RI, Korea Institute of Radiological and Medical Sciences, Seoul, Korea; and; 2 Radiological and Medico-Oncological Sciences, University of Science and Technology, Seoul, Korea

**Keywords:** microplastic, polystyrene, ^64^Cu, [^64^Cu]Cu-labeled polystyrene, PET

## Abstract

Plastics are used commonly in the world because of their convenience and cost effectiveness. Microplastics, an environmental threat and human health risk, are widely detected in food and consequently ingested. However, degraded plastics are found everywhere, creating an environmental threat and human health risk. Therefore, real-time monitoring of orally administered microplastics to trace them in the body is tremendously important. **Methods:** In this study, to visualize their absorption path, we labeled polystyrene with [^64^Cu]Cu-DOTA. We prepared radiolabeled polystyrene with ^64^Cu. Afterward, [^64^Cu]Cu-DOTA-polystyrene was orally administered to mice, and we evaluated its transit and absorption using PET imaging. The absorption path and distribution of [^64^Cu]Cu-DOTA-polystyrene were determined using PET over 48 h. Ex vivo tissue radio–thin-layer chromatography (TLC) was used to demonstrate the existence of [^64^Cu]Cu-DOTA-polystyrene in tissue. **Results:** PET images demonstrated that [^64^Cu]Cu-DOTA-polystyrene began to transit to the intestine within 1 h. Accumulation of [^64^Cu]Cu-DOTA-polystyrene in the liver was also observed. The biodistribution of [^64^Cu]Cu-DOTA-polystyrene confirmed the distribution of [^64^Cu]Cu-DOTA-polystyrene observed on the PET images. Ex vivo radio-TLC demonstrated that the detected γ-rays originated from [^64^Cu]Cu-DOTA-polystyrene. **Conclusion:** This study provided PET evidence of the existence and accumulation of microplastics in tissue and cross-confirmed the PET findings by ex vivo radio-TLC. This information may be used as the basis for future studies on the toxicity of microplastics.

## Visual Abstract

**Figure:**
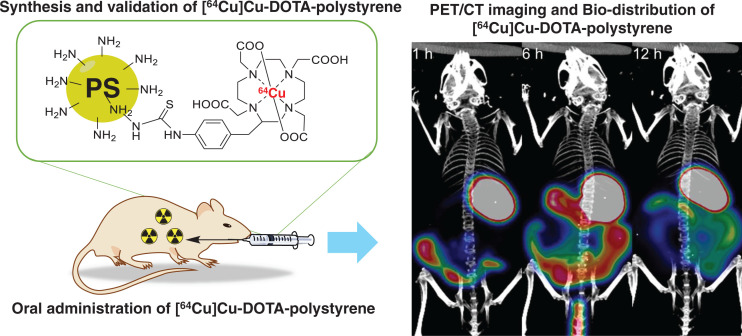

Microplastics with diameters of less than 5 mm are recognized as a new environmental threat and human health risk (1). Microplastics have been observed to accumulate in many different marine animals, including fish (*2*–*5*), copepods (*6*,*7*), mussels (*8*–*10*), European flat oysters (*11*), and others (*12*–*14*). Fiber-type microplastics have been found in mussels purchased at markets in Belgium (*15*). Considering that microplastics are widely detected in food, we can assume that microplastics are ingested along with the contaminated food. Therefore, it is highly likely that human consumption of microplastics is widespread. To understand the full significance of microplastic ingestion, the absorption path for microplastics ingested with foods needs to be visualized.

PET imaging is a powerful tool for observing absorption, distribution, metabolism, and excretion (*16*). PET can also be used to visualize the in vivo distribution of toxic substances labeled with radioactive isotopes, including diesel exhaust (*17*), and inhaled aerosols of toxic household disinfectants (*18*). Figure 1 shows a schematic of the study. We first identified the absorption path and distribution of microplastics using PET. Microplastic polystyrene was labeled with ^64^Cu ([^64^Cu]Cu, to yield [^64^Cu]Cu-DOTA-polystyrene) and then was orally administered to mice. In a separate experiment, ^64^Cu was orally administered as a control to assess the effects of the harsh stomach conditions on dechelated ^64^Cu. PET was performed to monitor the absorption and distribution of [^64^Cu]Cu-DOTA-polystyrene or ^64^Cu over 48 h. The ex vivo biodistributions of [^64^Cu]Cu-DOTA-polystyrene or ^64^Cu was measured. Ex vivo tissue radio–thin-layer chromatography (TLC) was performed to identify whether γ-rays emitted from the tissue originated from [^64^Cu]Cu-DOTA-polystyrene or from ^64^Cu.

**FIGURE 1.**
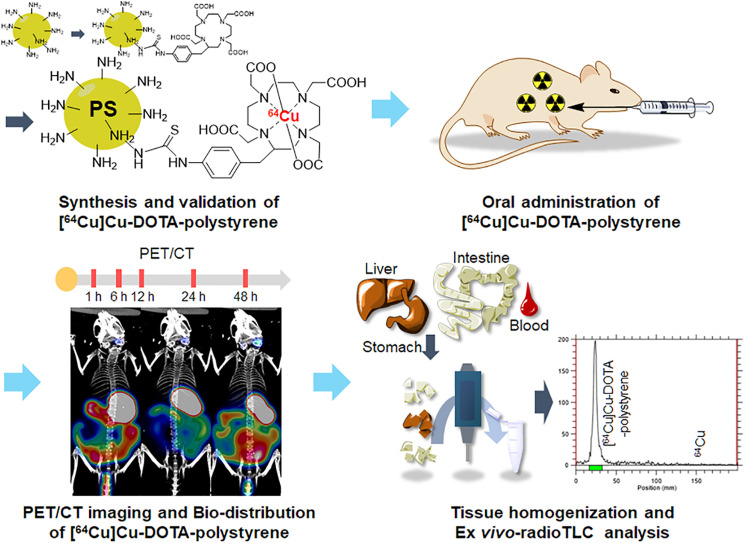
Schematic of experiment. [^64^Cu]Cu-DOTA-polystyrene was synthesized and validated using analytic instruments and radio-TLC. [^64^Cu]Cu-DOTA-polystyrene was orally administered to mice, and PET/CT was performed at 1, 6, 12, 24, and 48 h afterward. Tissues were weighed and counted at each time point for tissue distribution. Ex vivo radio-TLC assay was performed to determine whether detected γ-rays emitted from tissue originated from ^64^Cu or from [^64^Cu]Cu-DOTA-polystyrene. PS = polystyrene.

## MATERIALS AND METHODS

### Synthesis and Radiolabeling

To 300 µL of 0.1 M sodium carbonate buffer (pH 9.0), 2.5 mg of amino-polystyrene (0.2–0.3 µm; Spherotech) were added. Then, 260 µg (471.70 nmol) of *S*-2-(4-isothiocyanatobenzyl)-1,4,7,10-tetraazacyclododecane tetraacetic acid (*p*-SCN-Bn-DOTA) in 50 µL of deionized water were added, and the mixture (pH 9.0) was shaken at 1,000 rpm and 25°C for 20 h. Unconjugated *p*-SCN-Bn-DOTA was removed using an Amicon centrifugal filter (30-kDa cutoff; Millipore). DOTA conjugation was confirmed using Fourier-transform infrared spectroscopy (Nicolet iS5; Thermo Fisher Scientific), and the resulting spectra were analyzed using Omnic software from Nicolet Instrument Corp. To determine moles of DOTA per milligram of plastic, 50 µL of filtrate were analyzed by high-performance liquid chromatography (Waters). The quantity of DOTA in the filtrate was calculated from a standard curve (prepared from an analysis of known concentrations of DOTA). The conjugated moles of DOTA to polystyrene were then calculated by subtracting the moles of DOTA in the filtrate from the total moles of DOTA for the reaction. Physicochemical characterization of DOTA-polystyrene was performed using a field-emission scanning electron microscopy and dynamic light scattering. Concentrated DOTA-polystyrene was subsequently buffer-exchanged to isotonic buffered saline for subsequent radiolabeling. The final concentration before radiolabeling was 2.5 mg/100 µL.

Cyclotron-produced [^64^Cu]CuCl_2_ was dried and redissolved in 0.01 N HCl (final concentration, 9.25 MBq/µL). In a 1.5-mL tube, 155.4 MBq of [^64^Cu]CuCl_2_ were added to 80 µL of 0.1 M NaOAc buffer (pH 5). DOTA-polystyrene (2 mg in 80 µL) was added, and the mixture was shaken in a Thermomixer C (Eppendorf AG) at 40°C and 1,000 rpm for 30 min. ^64^Cu-labeled DOTA-polystyrene was purified using an Amicon centrifugal filter at 25°C, 3,000 rpm, for 30 min. By repeating this procedure, reaction buffer was exchanged to 1 × phosphate-buffered saline for further studies.

### In Vitro Stability Study

[^64^Cu]Cu-DOTA-polystyrene in phosphate-buffered saline (1.85 MBq/30 µL) was diluted in 270 µL of phosphate-buffered saline, hydrochloric acid-potassium chloride buffer (pH 2), human serum, or mouse serum. Each sample was incubated at 25°C (buffer) or 37°C (serum) for 48 h. Percentage stability was analyzed using instant TLC (0.1 M citric acid in water as a mobile phase).

### PET/CT

All animal experiments were performed under the institutional guidelines of the Korea Institute of Radiological and Medical Sciences. BALB/c nude mice (*n* = 5–7, 5 wk old; Shizuoka Laboratory Center) were used.

PET/CT images were acquired with an Inveon PET scanner (Siemens Medical Solutions). [^64^Cu]CuCl_2_ (4.81 MBq/100 µL) or [^64^Cu]Cu-DOTA-polystyrene (4.81 MBq/57.8 µg/100 µL) was orally administered to the mice. PET was performed at 1, 6, 12, 24, and 48 h afterward. The PET data were acquired for 15 min within 350–650 keV and were reconstructed using a maximum a priori with shifted Poisson distribution (SP-MAP) algorithm (target resolution 3). The voxel size was 0.776 × 0.776 × 0.796 mm. Regions of interest were drawn in the stomach, liver, and intestine using ASIpro (Siemens Medical Solutions) after coregistration of CT and PET images. SUV_max_ was then calculated.

### Biodistribution Study

The accumulated radioactivity concentration (percentage injected dose [%ID]/g) in each organ was measured at corresponding times after administration of [^64^Cu]Cu-DOTA-polystyrene or ^64^Cu.

### Ex Vivo Radio-TLC

Ex vivo radio-TLC assays were performed to determine whether the detected γ-rays emitted from the tissues were emitted from ^64^Cu or from [^64^Cu]Cu-DOTA-polystyrene at each time point. Homogenized samples were lysed in 10% sodium dodecyl sulfate phosphate-buffered saline (pH 7.4) instead of strong acid because low pH (≤1) induces dechelation of ^64^Cu from DOTA within 1 min (*19*). Similarly, a low pH in the stomach can disrupt stable chelation of [^64^Cu]Cu-DOTA, and this phenomenon was identified from ex vivo radio-TLC of the stomach at later time points.

### Statistical Analysis

The data are presented as the mean with SD. The Student *t* test was performed using Prism (version 5.0; GraphPad).

## RESULTS

### Synthesis and Radiolabeling

**FIGURE 2.**
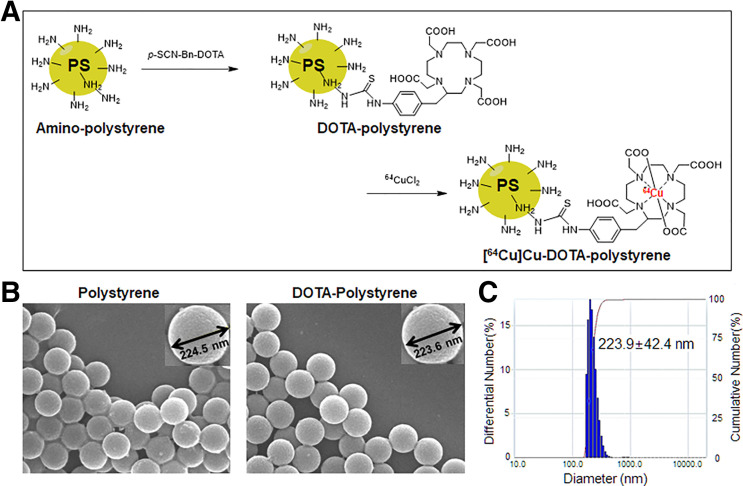
Synthesis of [^64^Cu]Cu-DOTA-polystyrene, and physicochemical validation of DOTA-polystyrene using field emission scanning electron microscopy and dynamic light scattering. (A) *p*-SCN-Bn-DOTA was conjugated to amine in polystyrene and labeled with ^64^Cu in pH 9 buffer. (B) Polystyrene particles and DOTA-polystyrene particles show no difference in field emission scanning electron microscopy results or dynamic light scattering. (C) No aggregation of DOTA-polystyrene particles occurred during conjugation. PS = polystyrene.

DOTA was conjugated by high-performance liquid chromatography and a Fourier-transform infrared spectrometer (Fig. 2A; Supplemental Fig. 1; supplemental materials are available at http://jnm.snmjournals.org). Per milligram of polystyrene, 184.78 ± 0.26 nmol of DOTA were conjugated. The particle size of polystyrene and DOTA-polystyrene was 223–224 nm, and no aggregation was observed (in either set of results) after the DOTA-conjugation reaction (Figs. 2B and 2C). The radiochemical yield of [^64^Cu]Cu-DOTA-polystyrene was 92.07% ± 3.20%, and radiochemical purity was 96.39% ± 1.66% (Supplemental Fig. 2A).

### In Vitro Stability Study

No significant dechelation was observed after 48 h in phosphate-buffered saline (96.34%), pH 2 (91.68%), human serum (93.23%), or mouse serum (96.83%). The in vitro stability study demonstrated that ^64^Cu-labeled polystyrene was stable for the period used in this study (Supplemental Fig. 2B).

### PET/CT

**FIGURE 3.**
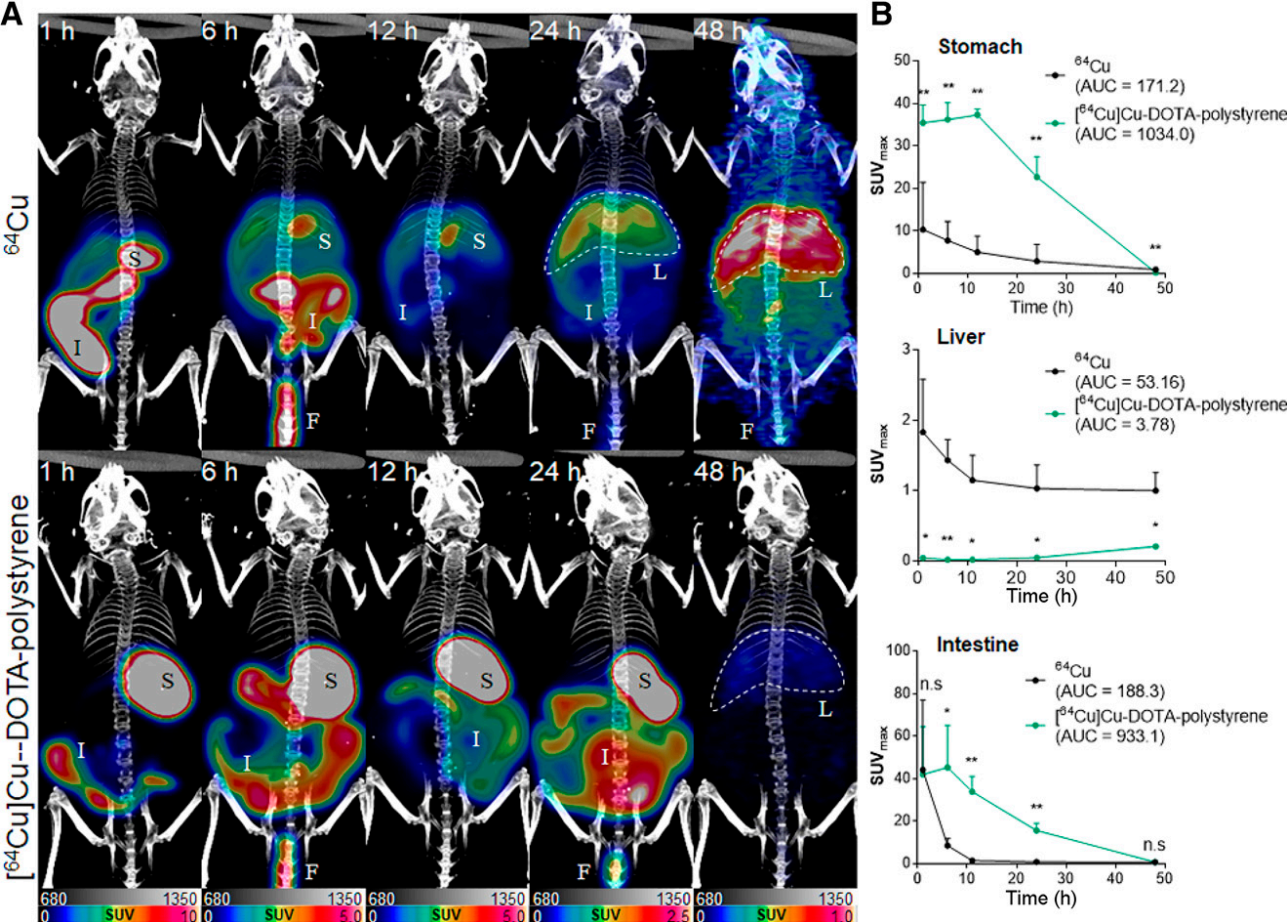
(A) Representative PET/CT of orally administered [^64^Cu]Cu-DOTA-polystyrene or ^64^Cu. [^64^Cu]Cu-DOTA-polystyrene accumulated in stomach and intestine for 24 h. Uptake of [^64^Cu]Cu-DOTA-polystyrene was observed in liver at 48 h after administration. However, ^64^Cu in stomach and intestinal track was rapidly cleared and transported to liver. (B) SUV_max_ of [^64^Cu]Cu-DOTA-polystyrene was significantly higher than that of ^64^Cu in stomach and intestine. In contrast, SUV_max_ of [^64^Cu]Cu-DOTA-polystyrene was significantly lower than that of ^64^Cu in liver. F = feces; I = intestine; L = liver; S = stomach. *n* = 5. *P < 0.05, Student *t* test. **P < 0.005, Student *t* test.

Figure 3 and Supplemental Figure 3 show the representative PET data at 1, 6, 12, 24, and 48 h after oral administration of [^64^Cu]Cu-DOTA-polystyrene or ^64^Cu. The corresponding time–activity curve is shown for the stomach, liver, and intestine. PET images demonstrate that [^64^Cu]Cu-DOTA-polystyrene remained in the stomach for up to 24 h. The SUV_max_ of [^64^Cu]Cu-DOTA-polystyrene in the stomach was 35.42 ± 4.25 at 1 h, 36.22 ± 3.91 at 6 h, 37.32 ± 1.34 at 12 h, 22.68 ± 4.81 at 24 h, and 0.20 ± 0.03 at 48 h. Polystyrene began its transit to the intestine within 1 h. The SUV_max_ of [^64^Cu]Cu-DOTA-polystyrene in the intestine was 41.93 ± 22.59 at 1 h, 45.29 ± 19.79 at 6 h, 33.84 ± 7.10 at 12 h, 15.59 ± 3.22 at 24 h, and 0.72 ± 0.75 at 48 h.

The in vivo absorption and distribution pattern of ^64^Cu on PET was statistically different at each PET measurement point (Fig. 3). The SUV_max_ in the stomach was 35.42 ± 4.25 for [^64^Cu]Cu-DOTA-polystyrene and 8.39 ± 6.98 for ^64^Cu at 1 h after administration. Compared with the SUV_max_ of ^64^Cu, the SUV_max_ of [^64^Cu]Cu-DOTA-polystyrene was 4.22-, 4.67-, 7.40-, and 7.83-fold greater in the stomach at 1, 6, 12, and 24 h, respectively. Moreover, the area under the curve (AUC) for [^64^Cu]Cu-DOTA-polystyrene was 6.03 times greater in the stomach (AUC for [^64^Cu]Cu-DOTA-polystyrene, 1,034.0; AUC for ^64^Cu, 171.2).

The SUV_max_ in the intestine was 45.29 ± 19.75 for [^64^Cu] Cu-DOTA-polystyrene and 8.40 ± 3.36 for ^64^Cu at 1 h after administration. Compared with the SUV_max_ of ^64^Cu, the SUV_max_ of [^64^Cu]Cu-DOTA-polystyrene was 5.38-, 23.26-, and 19.43-fold greater in the intestine at 6, 12, and 24 h, respectively. Moreover, the AUC for [^64^Cu]Cu-DOTA-polystyrene was 4.95 times greater in the intestine (AUC for [^64^Cu]Cu-DOTA-polystyrene, 933.1; AUC for ^64^Cu, 188.3).

The SUV_max_ in the liver was 0.04 ± 0.03 for [^64^Cu]Cu-DOTA-polystyrene and 1.83 ± 0.75 for ^64^Cu at 1 h after administration. Compared with the SUV_max_ of ^64^Cu, the SUV_max_ of [^64^Cu]Cu-DOTA-polystyrene was 0.02-, 0.01-, 0.01-, and 0.04-fold lower in the liver at 1, 6, 12, and 24 h, respectively. Moreover, the AUC for [^64^Cu]Cu-DOTA-polystyrene was 0.07 times lower in the liver (AUC for [^64^Cu]Cu-DOTA-polystyrene, 3.78; AUC for ^64^Cu, 53.16). PET showed a higher uptake of ^64^Cu in the liver because ^64^Cu was largely adsorbed by albumin and transcuprein and then carried to the liver (*20*). Both [^64^Cu]Cu-DOTA-polystyrene and ^64^Cu were partly excreted in feces.

### Biodistribution Study

**FIGURE 4.**
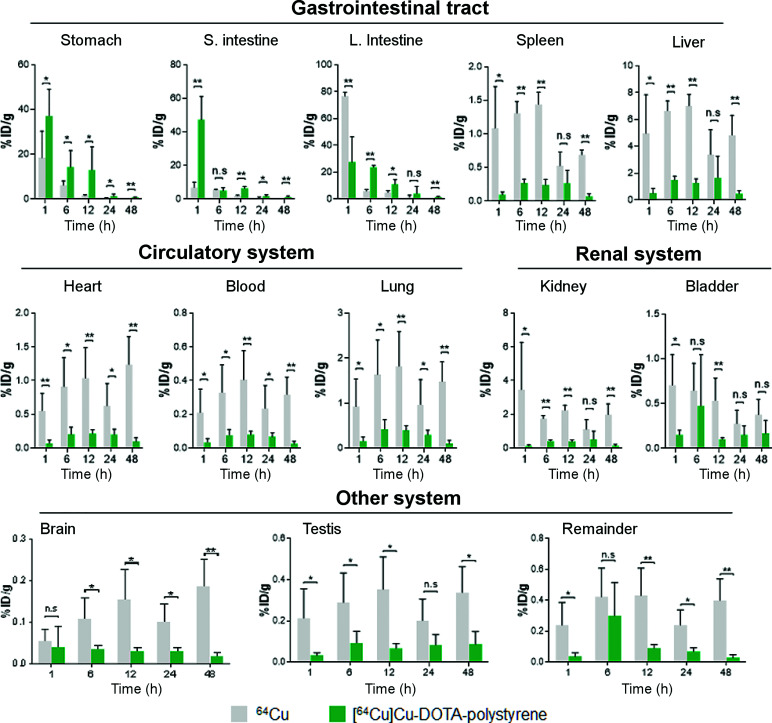
Biodistribution results for ^64^Cu and [^64^Cu]Cu-DOTA-polystyrene in gastrointestinal tract (stomach, intestine, and liver), circulatory organs (heart, lung, and blood), renal system (kidney and bladder), and brain. Overall, accumulation pattern of biodistribution was similar to that of SUV in PET images. %ID/g of [^64^Cu]Cu-DOTA-polystyrene in stomach, small intestine, and large intestine was significantly higher than that of ^64^Cu. However, in liver, %ID/g of [^64^Cu]Cu-DOTA-polystyrene was lower than that of ^64^Cu. Additionally, [^64^Cu]Cu-DOTA-polystyrene transited to gastrointestinal tract (liver and spleen), circulatory system (heart, blood, and lung), renal system (kidney and bladder), and even to brain and testis. In contrast, most ^64^Cu accumulated in large intestine, stomach, and small intestine at 1 h after administration. Subsequently, ^64^Cu transited quickly to other organs, including liver. %ID/g in all other organs tested, including liver, spleen, heart, blood, lung, kidney, bladder, brain, and testis, was greater for ^64^Cu than for [^64^Cu]Cu-DOTA-polystyrene. *n* = 5. *P < 0.05, Student *t* test. **P < 0.005 Student *t* test. n.s. = not statistically significant.

Figure 4 shows the biodistribution results for the organs of interest. Overall, the accumulation pattern was similar to that of SUV in the PET images. In the stomach, the %ID/g of [^64^Cu]Cu-DOTA-polystyrene was 2.01-, 2.31-, 8.28-, 3.61-, and 13.27-fold greater than that of ^64^Cu at the 1-, 6-, 12-, 24-, and 48-h time points, respectively. In the small intestine, the %ID/g of [^64^Cu]Cu-DOTA-polystyrene was 6.89-, 0.92-, 3.44-, 2.50-, and 11.44-fold greater than that of ^64^Cu at the 1-, 6-, 12-, 24-, and 48-h time points, respectively. In the large intestine, the %ID/g of [^64^Cu]Cu-DOTA-polystyrene was 0.36-, 3.95-, 2.28-, 3.11-, and 13.75-fold greater in the stomach that that of ^64^Cu at the 1-, 6-, 12-, 24-, and 48-h time points, respectively. In the liver, the %ID/g of [^64^Cu]Cu-DOTA-polystyrene was 0.10-, 0.22-, 0.18-, 0.49-, and 0.10-fold lower than that of ^64^Cu at the 1-, 6-, 12-, 24-, and 48-h time points, respectively. Additionally, we observed transit of [^64^Cu]Cu-DOTA-polystyrene to the liver, spleen, heart, blood, lung, kidney, bladder, and testis.

In contrast, most of the ^64^Cu accumulated in the large intestine, stomach, and small intestine at 1 h after administration. ^64^Cu then quickly transitioned to other organs, including the liver. The %ID/g was greater for ^64^Cu than for [^64^Cu]Cu-DOTA-polystyrene in all other organs, including the liver (9.59-fold), spleen (12.0-fold), heart (7.85-fold), blood (5.83-fold), lung (25.69-fold), kidney (26.92-fold), bladder (1.35-fold), brain (1.36-fold), and testis (6.35-fold).

### Ex Vivo Radio-TLC

The ex vivo radio-TLC assay results for other tissues (liver, small, and large intestine) demonstrated that the radiation signal was from [^64^Cu]Cu-DOTA-polystyrene, not from ^64^Cu (Supplemental Fig. 4).

## DISCUSSION

We first identified the in vivo distribution of microplastics in mice by labeling microplastic polystyrene with the radioisotope ^64^Cu, orally administering [^64^Cu]Cu-DOTA-polystyrene (radiolabeled microplastic polystyrene) to mice, and using PET to trace its absorption and distribution. Next, ex vivo biodistribution studies confirmed [^64^Cu]Cu-DOTA-polystyrene accumulation in specific organs. Ex vivo radio-TLC was used to confirm that the detected γ-rays originated from [^64^Cu]Cu-DOTA-polystyrene. Exposure to microplastics in food and water through oral administration is a significant environmental and health problem (*21*–*23*). However, it is extremely likely that microplastics are widely distributed within the food we eat.

The advantage of PET is that it is possible to observe the in vivo absorption, distribution, metabolism, and excretion of substances labeled with radioactive isotopes without killing the animal (*16*). Although fluorescence is commonly used for in vivo exposure and biodistribution studies, fluorescence in animal bodies can be absorbed by bone and soft tissues, and prolonged exposure to ultraviolet light can result in bleaching and loss of fluorescence intensity (*24*). Therefore, quantification of fluorescent images is limited, compared with PET images. In addition, when microplastic-conjugated fluorescent dyes are used, animals must be killed to observe the absorption and accumulation of the microplastics over time. [^64^Cu]Cu-DOTA-polystyrene transit and absorption were observed within the same animal using PET, without killing the animal.

In this study, we first observed the in vivo pathways (absorption, distribution, metabolism, and excretion) of microplastics labeled with a radioisotope using PET. To trace the polystyrene after oral administration, we selected ^64^Cu and *p*-SCN-Bn-DOTA for the radiolabeling of plastic particles. We subsequently confirmed that the detected radiation was emitted from the [^64^Cu]Cu-DOTA-polystyrene, not from ^64^Cu, using ex vivo radio-TLC. DOTA-*N*-hydroxysuccinimide ester and *p*-SCN-Bn-DOTA are frequently used chelators (*19*). DOTA conjugation was confirmed by Fourier-transform infrared spectroscopy, because the functional groups of DOTA show specific bands (Supplemental Fig. 1).

The biodistribution study also demonstrated that the distribution of [^64^Cu]Cu-DOTA-polystyrene was different from that of ^64^Cu. The biodistribution study provided quantification of [^64^Cu]Cu-DOTA-polystyrene accumulation in each organ, even at low levels of emitted γ-rays. Using the biodistribution, we observed the transit and accumulation of [^64^Cu]Cu-DOTA-polystyrene within the gastrointestinal tract (stomach, intestine, and liver), circulatory organs (heart, lung, and blood), renal system (kidney and bladder), and even brain, at 1 h after oral administration.

In contrast, orally administered ^64^Cu was rapidly removed from the stomach, small intestines, and large intestine, before transit to the other organs, including the liver (Fig. 4). We also observed a higher SUV in the liver on PET for the group that was orally administered ^64^Cu (Fig. 3). In a previous report, accumulation of ^64^Cu in the liver was observed on PET (*20*). For kidney and spleen, the levels of ID/g (1 < %ID/g < 10) at 1 h were 3.47 and 1.08, respectively. For bladder, testis, heart, lung, and blood, the levels of ID/g (%ID/g < 1) at 1 h were 0.70, 0.22, 0.55, 0.92, and 0.21, respectively. The rapid distribution of orally administered ^64^Cu to the other organs may have occurred because digestive fluid may facilitate solubilization of ^64^Cu in the stomach. ^64^Cu was partly cleared in feces after transit through the gastrointestinal tract, and the remaining ^64^Cu was distributed to other organs, including the liver.

In mice, the normal gastric pH is approximately 3.0 (*25*). During transit through the stomach, [^64^Cu]Cu-DOTA-polystyrene may encounter harsh conditions, possibly dechelating ^64^Cu. However, our ex vivo radio-TLC assay—through comparison data between ^64^Cu and [^64^Cu]Cu-DOTA-polystyrene—ensured that there was no dechelation of ^64^Cu in the stomach or liver at 1 h. According to the data, the detected signal from PET and the biodistribution at 1 h in all other organs, including the liver, was from [^64^Cu]Cu-DOTA-polystyrene, not from dechelated ^64^Cu. Although the acidity of the stomach did affect dechelation at 6 h after administration, the other organs were not influenced by dechelation of ^64^Cu from radio-TLC (Supplemental Fig. 4). Therefore, each data point obtained from PET and biodistribution was confirmed with [^64^Cu]Cu-DOTA-polystyrene. Consequently, the dechelation of ^64^Cu could be negligible (Fig. 4).

Recently, several animal studies have been published on the effects of microplastics (*26*–*29*). Microplastic ingestion may induce behavioral disorders in mice (*30*). Therefore, it is important to observe how microplastics are distributed in the body after ingestion. Remarkably, biodistribution demonstrated that [^64^Cu]Cu-DOTA-polystyrene was distributed to all tested organs, including the testis, even after a one-time single dose. Thus, [^64^Cu]Cu-DOTA-polystyrene may transit and accumulate in all organs even 1 h after oral administration. According to a recent report, a 4-wk exposure to polystyrene (1.0% w/v, 10 mL) induced male reproductive dysfunction in mice (*31*). On the basis of that mouse study and our present results, we assumed that at least 4 wk of polystyrene exposure may induce hazardous effects on individual organs such as digestive organs, circulatory organs, and excretory organs.

We used BALB/c nude mice because we aimed to assess the tumorigenesis after longitudinal polystyrene exposure for further study. When different strains of mouse were used, possibly different degrees of absorption, distribution, metabolism, and elimination of polystyrene might be observed during PET.

The polystyrene used in these experiments was surface-coated with amines, and it seems likely that this process might affect their biodistribution. Polystyrene is a highly hydrophobic particle, and the addition of multiple primary amines (hydrophilic and positively charged at physiologic pH) and DOTA chelators (hydrophilic and negatively charged at physiologic pH) on the surface may influence biodistribution. Hydrophobic compounds and aggregates tend to show uptake and retention in the liver, and uptake in the liver may therefore be influenced by the surface modifications. Even if radiotracers were prepared from the same material, differences in size, shape, and surface charge can affect biodistribution and clearance. Generally, small nanoparticles penetrate capillary walls more easily than large nanoparticles, and positively charged nanoparticles are cleared more quickly by macrophages (*32*–*34*). Smaller and negatively charged silica nanoparticles have enhanced intestinal permeation by opening tight junctions (*35*). In this study, we selected a sphere-shaped and 0.2-µm–sized polystyrene and observed no significant differences in size or shape after DOTA conjugation. ^64^Cu-labeled DOTA-polystyrene contains uncoordinated carboxylic acids, which have negative charges, and free amines, which have positive charges at physiologic pH. These surface charges may affect the permeability of the gastrointestinal tract and distribution. Recent fluorescence-conjugated microplastic studies indicated that the biodistribution of microplastics was dependent on the size of the particles (*26*,*36*). According to the result of Deng et al. (*26*), the accumulation in the kidney and gut was greater for 5-µm microplastics than for 20-µm microplastics. Therefore, it is possible that a smaller amount of radioisotope-labeled microplastics might accumulate in mouse organs when larger microplastics are used.

## CONCLUSION

Our results demonstrate the utility of PET for visualizing the absorption and distribution of polystyrene microplastics radiolabeled with ^64^Cu. PET provides information on the accumulation of microplastics in vivo and can provide information on how each organ might be affected after continuous microplastic exposure. The biologic effects of long-term exposure to microplastics in each organ affected in this study will be evaluated in future studies.

## DISCLOSURE

This study was funded by the Ministry of Science and ICT (MSIT), Republic of Korea (NRF-2020R1F1A1061476, 2021M2E8A1039980, 50536-2021, and 50461-2021). No other potential conflict of interest relevant to this article was reported.

## Notes

KEY POINTS **QUESTION:** What is the absorption path for the microplastics ingested with foods we eat? **PERTINENT FINDINGS:** PET images demonstrated that [^64^Cu]Cu-DOTA-polystyrene accumulated in the stomach and began to transit to the intestine within 1 h. Accumulation of [^64^Cu]Cu-DOTA-polystyrene in the liver was also observed. **IMPLICATIONS FOR PATIENT CARE:** On the basis of the observed in vivo accumulation of [^64^Cu]Cu-DOTA-polystyrene, we can assume that long-term exposure to polystyrene may be a potential risk to human health.
